# Frequency and Types of Periapical Radiographic Lesions Associated With Pulpitis in a Tertiary Care Hospital

**DOI:** 10.7759/cureus.42529

**Published:** 2023-07-27

**Authors:** Arzoo Fatima, Ushwa N Khan, Amara Nazir, Mobeen Akhtar, Sadiq Amin Ahmed Rana, Muhammad Kashif

**Affiliations:** 1 Operative Dentistry, Bakhtawar Amin Medical and Dental College, Multan, PAK; 2 Operative Dentistry, Nishtar Institute of Dentistry (NID), Multan, PAK; 3 Oral Pathology, Bakhtawar Amin Medical and Dental College, Multan, PAK

**Keywords:** pulpitis, periapical granuloma, periapical cyst, periapical view radiograph, peri-apical lesion

## Abstract

Objective

This cross-sectional study aimed to determine the frequency and types of periapical radiographic lesions (PARLs) associated with pulpitis in patients visiting a tertiary care hospital.

Methodology

A sample of patients diagnosed with pulpitis, aged 18 years or older, was recruited following a convenient sampling technique. Clinical examinations were conducted to confirm the diagnosis, and radiographic evaluations, including periapical (PA), occlusal, orthopantomogram (OPG), and cone beam computed tomography (CBCT) radiographs were obtained. The radiographs were evaluated for the presence of PA lesions, and the type, size, and location of the lesions were documented. Data were analyzed using IBM SPSS Statistics for Windows, Version 25.0 (IBM Corp., Armonk, NY, USA).

Results

A total of 120 patients (equal gender ratio) with a mean age of 32.6 ± 6.39 years participated in the study. PA views were the most frequently performed radiographic view (86.7%), followed by occlusal views (8.3%). The most prevalent radiographic lesion was the *widening of the periodontal ligament (PDL) space* (34.2%), followed by *PA granuloma* (17.5%) and *PA cyst* (10.8%). The most frequently encountered diagnosis was *pulpitis *(51.7%), followed by *irreversible pulpitis with apical periodontitis* (25.8%). Education level, swelling, pus discharge, medicine history, and tooth wear showed statistically significant associations (*P* ≤ 0.05) with the variables under investigation.

Conclusions

The most common lesions observed were *widening of the PDL space*, *PA granuloma*, and *PA cyst*. The findings contribute to the local epidemiological and clinical data, enriching the existing database. Understanding the prevalence and characteristics of PA lesions associated with pulpitis can aid in accurate diagnosis and treatment planning for patients with pulpal pathologies.

## Introduction

Dental caries and its subsequent condition, pulpitis, are among the most frequent complaints for which patients seek dental care. The dental pulp, a sterile connective tissue, is safeguarded by enamel, dentin, and cementum. Severe damage to the pulp chamber leads to inflammation, pain, pulpal necrosis, and potential periapical (PA) pathologies. PA radiolucency commonly arises due to trauma, caries, or tooth wear and can indicate the presence of a cyst or granuloma, discernible through radiographic evidence. Various types of PA radiographic lesions (PARLs), such as cysts, granulomas, and abscesses, have been identified [1].

Pulpal inflammation, referred to as reversible pulpitis, typically occurs when the underlying cause, such as defective restorations or caries, is removed. It is characterized by pathological inflammation of the pulp tissue and may initially be asymptomatic. Reversible pulpitis commonly manifests at sites of recent trauma or defective restorations in both primary and permanent teeth. Diagnostic criteria include pain from a cold test that lasts no longer than 30 seconds, lack of percussion sensitivity, absence of spontaneous pain, and no heat sensitivity [2]. On the other hand, irreversible pulpitis is characterized by severe, medication-relieved pain lasting from minutes to hours and associated with PA involvement, such as PA cysts or granulomas. It occurs when the pulp tissue becomes necrotic or partially necrotic. Radiographs may reveal a widening of the periodontal ligament (PDL) space at the tooth apex, while vitality tests generally yield negative results. As the inflammatory process progresses from the root canal to PA tissue, positive percussion is observed [3]. Chronic apical periodontitis can give rise to PA cysts (7.5 mm or larger) or PA granulomas (7.4 mm or smaller) due to a shift in the host immune response [4].

Accurate diagnosis of pulpitis requires determining the etiology, nature, and intensity of pain through the use of electric pulp testing, thermal tests, and mechanical tests. Additional investigations, such as PA view radiographs, are performed to diagnose irreversible pulpitis [5]. Although PA radiographs combined with clinical examination have long been the standard for endodontic diagnosis and postoperative evaluation, they provide a two-dimensional (2D) view of three-dimensional (3D) structures. While PA radiographs are commonly used to assess the status of the PA area, orthopantomogram (OPG), a panoramic radiograph of the mandible, maxilla, and teeth, is useful for evaluating general dental health, identifying caries or pulp-related diseases, assessing infections (sinusitis, periodontitis, and PA abscesses), and detecting tumors and cysts. OPGs are frequently employed in dental practice and occasionally in the emergency department, offering a convenient, cost-effective, and rapid means of evaluating jaw anatomy and related pathologies [6,7]. Cone beam computed tomography (CBCT), which aids in the differential diagnosis of apical periodontitis, and magnetic resonance imaging (MRI) should be used sparingly to minimize radiation exposure. Clinicians should consider CBCT imaging only when lower dose conventional dental radiography or alternative imaging modalities cannot provide sufficient diagnostic information [8].

Once a proper diagnosis has been established, the appropriate treatment may involve endodontic treatment followed by restoration [9]. Surgical procedures, such as PA surgery, corrective surgery, intentional replantation, and root removal, are commonly performed for peri-radicular conditions. The primary objective of this study was to assess the frequency and prevalence of patients with pulpitis and associated PARLs, such as granulomas, cysts, and abscesses. These findings will contribute to the local epidemiological and clinical data in our region, enriching the existing database.

## Materials and methods

Study design

The current study was conducted in the Department of Operative Dentistry and the Department of Oral Pathology, Bakhtawar Amin Medical and Dental College, Multan, from January 2023 to May 2023. The study adopted a cross-sectional design to investigate the frequency and type of PARLs associated with irreversible pulpitis in patients who visited a tertiary care hospital. Ethical approval was obtained (No. 65/2023/COD) from the Institutional Research Board (IRB) of Bakhtawar Amin Dental College and Hospital, to ensure compliance with ethical guidelines. Patient confidentiality and informed consent were strictly maintained throughout the study.

Sample selection

A convenient sampling technique was followed for the selection of study subjects. The sample selection involved defining inclusion and exclusion criteria. Inclusion criteria encompassed patients with clinically diagnosed pulpitis, aged 18 years or older, and those visiting the tertiary care hospital. Exclusion criteria included patients with systemic diseases or conditions that could affect the pulp status, those who had received root canal treatment or endodontic surgery, and those with missing radiographic records. The sample size was determined based on statistical calculations and feasibility considerations.

Data collection

The World Health Organization (WHO) sample size calculator was used for determining the sample size. Data collection began with patient recruitment. One hundred and twenty eligible patients were identified based on the inclusion and exclusion criteria, and the purpose and procedures of the study were explained to potential participants. Written informed consent was obtained from patients who agreed to participate. Clinical examinations were then conducted to confirm the diagnosis of pulpitis, and relevant clinical parameters such as pain intensity, tooth vitality, and periapical tenderness were recorded. The visual analog scale (VAS) was used to determine the intensity of pain, and it was categorized as *mild*, *moderate*, and *severe*. The common pain-radiating sites of pulpitis, including the upper jaw, temporal region, ipsilateral face, neck, and shoulder, were noted in history.

The radiographic evaluation involved obtaining PA, OPG, occlusal view, and CBCT radiographs for the selected teeth using standard radiographic techniques. The radiographs were ensured to be of high quality and properly exposed. An experienced endodontist evaluated the radiographs for the presence of periapical lesions. The type, size, and location of any detected periapical lesions were carefully documented.

All data collected during the study were recorded using a structured data collection form to ensure accuracy and consistency. Standardized terminology and definitions were used during the data recording process. Patient data were anonymized by assigning unique identifiers or codes to maintain confidentiality.

Statistical analysis

The collected data were analyzed using IBM SPSS Statistics for Windows, Version 25.0 (IBM Corp., Armonk, NY, USA). The frequency and percentage of periapical lesions associated with pulpitis were calculated. The distribution of lesion types, such as periapical granuloma or periapical cyst, within the sample was also determined. The chi-square and Fisher's exact tests were used to determine the association between the type of radiographic diagnosis with other demographic, clinical, and radiographic variables. A *P*-value of ≤0.05 was considered statistically significant. The findings were interpreted in light of existing literature and theories. The clinical implications and potential significance of the observed frequency and types of periapical lesions were discussed. Limitations and potential biases of the study, such as selection bias or the use of radiographic examination alone, were considered. The results were compared with similar studies, and any discrepancies or similarities were discussed.

## Results

**Figure 1:**
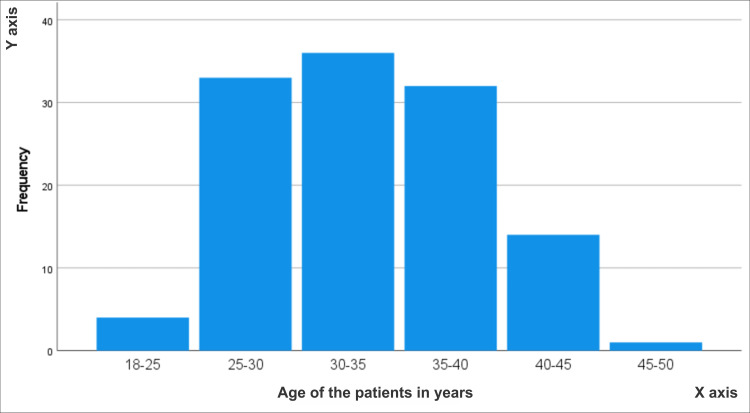
Histogram showing the distribution of the study population according to age groups.

In this study, the average age of the patients was found to be 32.6 ± 6.39 years. Figure 1 demonstrates that among the 120 patients included in the study, the majority fell within the age range of 25 to 40 years. Furthermore, the study population consisted of an equal number of male and female subjects, with 60 males and 60 females, resulting in a gender ratio of 1:1.

**Figure 2:**
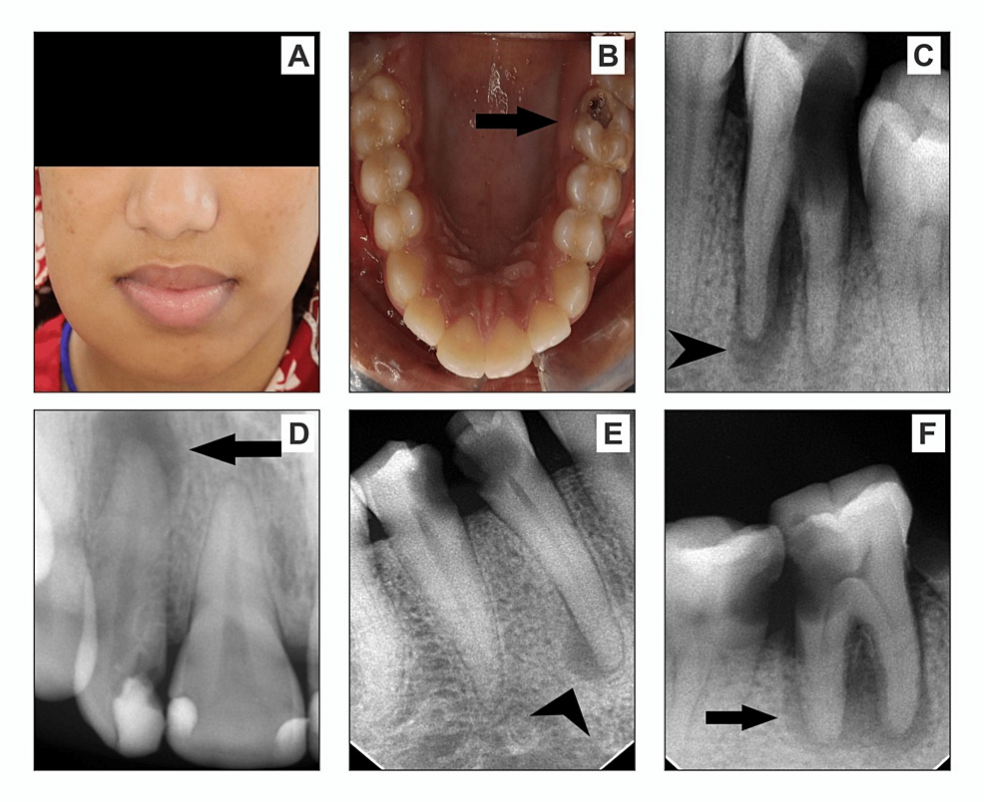
Clinical and radiographic findings of patients with periapical pathologies: (A) 19-year-old female patient presented with swelling and pain, (B) grossly carious left upper first molar with irreversible pulpitis, (C) lower first molar with carious crown and radiolucent lesion involving full length of mesial root, (D) upper lateral incisor with recurrent caries and periapical cyst, (E) lower first premolar with periapical granuloma and second premolar with the widening of the PDL space, (F) lower second molar with characteristic endo-perio lesions. PDL, periodontal ligament

PA views were the most frequently performed radiographic view, comprising 86.7% of the total. The second most common radiographic view was occlusal views, accounting for 8.3% of the total. OPG and CBCT were performed less frequently, with each representing 2.5% of the total. The most prevalent radiographic lesion was the *widening of the PDL space*, accounting for 34.2% of the cases. *PA granuloma* was the second most commonly observed lesion, comprising 17.5% of the cases. *PA cyst* was the third most frequent lesion, representing 10.8% of the cases. *Root resorption* was detected in 5.8% of the cases. Lesions *extending to adjacent teeth* are found in 2.5% of the cases. A significant portion of cases (28.5%) exhibited a combination of *widening of the PDL space* and *PA granuloma* without root resorption, indicating a frequent co-occurrence of these lesions. Conversely, only 0.8% of the cases demonstrate a combination of *widening of the PDL space* and *PA granuloma* with root resorption, suggesting a less common association (Figure 2).

The most frequently encountered diagnosis was *pulpitis*, accounting for 51.7% of the cases. The second most common diagnosis was *irreversible pulpitis with apical periodontitis*, representing 25.8% of the cases. *Necrosis* was observed in 5% of the cases, while *apical cyst *was diagnosed in 3.3% of the cases. *Apical granuloma* was seen in 14.2% of the cases (Table 1).

**Table 1:** Frequency of radiographic views performed, types of radiographic PA lesions associated, and final diagnoses of patients presented in the dental outpatient department. PDL, periodontal ligament; PA, periapical; OPG, orthopantomogram; CBCT, cone beam computed tomography

Variables	Frequency (Percentage)
Investigations/type of radiographic views	
PA view	104 (86.7%)
Occlusal view	10 (8.3%)
OPG	03 (2.5%)
CBCT	03 (2.5%)
Types of radiographic lesions	
Widening of the PDL space	41 (34.2%)
PA granuloma	21 (17.5%)
PA cyst	13 (10.8%)
Root resorption	7 (5.8%)
Extending to adjacent teeth	3 (2.5%)
Widening of the PDL space and PA granuloma without root resorption	34 (28.5%)
Widening of the PDL space and PA granuloma with root resorption	1 (0.8%)
Diagnosis	
Pulpitis (reversible and irreversible pulpitis)	62 (51.7%)
Irreversible pulpitis with apical periodontitis	31 (25.8%)
Necrosis	6 (5%)
Apical cyst (7.5 cm and above)	4 (3.3%)
Apical granuloma (7.4 mm )	17 (14.2%)

The education levels of the participants were categorized into three groups: *no formal education*, *less than 10 years of education*, and *more than 10 years of education*. Out of the total 120 participants, 20 (16.7%) had no formal education, 65 (54.2%) had less than 10 years of education, and 35 (29.2%) had more than 10 years of education. Statistical analysis was performed to examine the relationship between education levels and the variables under investigation. The results indicated a statistically significant association between education and the type of PA lesions, with a *P*-value of 0.026. This suggests that education level has a significant impact on the variables studied in this research.

Out of the total 120 participants, 3 (2.5%) reported having swelling, while the majority (117, 97.5%) did not report any swelling. A strong statistically significant association existed between the presence of swelling and the type of PA lesion, with a *p*-value of 0.001. The presence of pus discharge was assessed among the study participants. The results showed that out of the total 120 participants, 1 (0.8%) reported having pus discharge, while the majority, 119 (99.2%), did not report any pus discharge. Statistical analysis was conducted to examine the relationship between the presence of pus discharge and the variables of interest. The analysis revealed a statistically significant association between the presence of pus discharge and the variables, with a *P*-value of 0.033.

The presence of tooth wear was also assessed among the study participants. The results indicated that out of the total 120 participants, 21 (17.5%) reported having tooth wear, while the majority (99, 82.5%) did not report any tooth wear. Statistical analysis was conducted to examine the relationship between the presence of tooth wear and the variables of interest. The analysis revealed a statistically significant association between the presence of tooth wear and the variables, with a *P*-value of 0.001. This suggests that the presence of tooth wear is significantly related to the variables examined in this study.

In summary, several variables in the study, including the level of education, swelling, pus discharge, medication history, and tooth wear, were found to be significantly associated with the final diagnoses. On the other hand, variables such as gender, occupation, pain, past treatment history, number of carious teeth, tooth trauma, and tooth discoloration did not show a significant association with the final diagnoses, as indicated in Table 2.

**Table 2:** Association of demographic and clinical characteristics of patients with the type of periapical lesions.

Variables	Frequency (percentage)	*P*-value
Gender		0.299
Male	60 (50.0%)	
Female	60 (50.0%)	
Education		0.026
No formal education	20 (16.7%)	
Less than 10 years of education	65 (54.2%)	
More than 10 years of education	35 (29.2%)	
Occupation		0.472
Employed	33 (27.5%)	
Laborers	10 (8.3%)	
Housewives	23 (19.2%)	
Unemployed	31 (25.8%)	
Students	22 (19.16%)	
Pain		0.957
No	17 (14.2%)	
Yes	103 (85.8%)	
Swelling		<0.001
Yes	3 (2.5%)	
No	117 (97.5%)	
Pus discharge		0.033
Yes	1 (0.8%)	
No	119 (99.2%)	
Medication history		0.02
Yes	49 (40.8%)	
No	71 (59.2%)	
Past treatment history		0.421
Yes	32 (26.7%)	
No	88 (73.3%)	
Number of carious teeth		0.621
1	27 (22.5%)	
2	36 (30%)	
3	34 (28.3%)	
4	21 (17.5%)	
>4	2 (1.7%)	
Tooth trauma		0.28
Yes	7 (5.8%)	
No	113 (94.2%)	
Discolored tooth		0.149
Yes	12 (10%)	
No	108 (90%)	
Tooth wear		0.001
Yes	21 (17.5%)	
No	99 (82.5%)	

Based on the cross-tabulation analysis of radiographic lesions and various diagnoses, the results indicate that the widening of PDL on PA radiographs was observed among 41 (34.16%) cases, and the majority of patients (93, 77.5%) were affected by pulpitis (reversible/irreversible) and irreversible pulpitis with apical periodontitis. These results indicate a significant association between the presence of radiographic lesions and different diagnoses (*P* = 0.003). The findings emphasize the importance of radiographic evaluation in diagnosing and managing dental pulp and PA conditions (Table 3).

**Table 3:** Association of the type of radiographic lesions with final diagnosis.

		Diagnosis					Total
		Pulpitis (reversible, irreversible)	Irreversible pulpitis with apical periodontitis	Necrosis	Apical cyst (7.5 mm and above)	Apical granuloma (7.4 mm)	
Types of radiographic lesions	Widening of PDL	20	17	4	0	0	41
	Periapical granuloma	4	0	0	0	17	21
	Periapical cyst	5	5	0	3	0	13
	Root resorption	4	1	2	0	0	7
	Extending to adjacent teeth	2	1	0	0	0	3
	Widening of PDL and periapical granuloma with root resorption	26	7	0	1	0	34
	Widening of PDL and periapical granuloma without root resorption	1	0	0	0	0	1
Total		62	31	6	4	17	120

## Discussion

The presence of PA lesions associated with pulpitis holds significant clinical importance, as the majority of patients presenting with pulpitis also exhibit PA radiolucency, cysts, granulomas, or abscesses. Treating these conditions through endodontic procedures is a complex and challenging process. PA lesions often result in necrotic dental pulp and are among the most prevalent pathological conditions affecting the alveolar bone [10]. To consolidate the search for PA lesions and ensure reproducibility, our research strategy used the terms *PA lesions* in association with *pulpitis*. We diagnosed 120 patients with PA lesions, including cysts or granulomas, who were confirmed to have pulpitis.

Regarding the prevalence of PA lesions associated with pulpitis, our study found that the average age of patients was 32.6 ± 6.39 years, with the majority falling within the age range of 25 to 40 years. In a study conducted in Japan with 244 patients, the average age was 52.6 ± 14.9 years, and the majority fell within the age range of 29 to 50 years. In the French population, the prevalence of PARLs was highest among patients with an average age of 56.7 ± 16.1 years and within the age group of 40 to 50 years. The Belarusian population showed the highest values, with an average age of 46.8 ± 26 years and an age group of 45 to 49 years. In contrast, the Norwegian population had the lowest values, with the age group of 46 to 50 years, showing a lower incidence of PARLs [11]. These variations in incidence can be attributed to differences in awareness and access to advanced facilities. The advanced countries of France and Belarus have higher incidence rates in later age groups due to better amenities compared to our region, whereas Norway exhibits a similar lower incidence rate in the age group of 45 to 50 years [9]. Additionally, our study revealed that the majority of patients had less than 10 years of formal education, whereas the Russian population had a mean of approximately 10 years of formal education. Japan, being technologically advanced, also has a more educated population, with 10 or more years of education, resulting in a lower incidence rate of PARLs compared to our region. The lower literacy rate of 58.9% in Pakistan, compared to Japan's 99%, contributes to the lack of awareness among the population regarding PARLs associated with pulpitis, leading to a higher prevalence of the condition [12].

Pain was a prevalent symptom among the study participants, with 103 out of 120 patients experiencing pain. In contrast, only 17 patients presented without pain. Comparatively, a study conducted in Russia included 150 patients who underwent routine check-ups, resulting in a lower incidence of pain. Lack of awareness and absence of routine check-ups contribute to the different incidence rates observed in our region compared to Russia [13]. The intensity of symptoms was assessed and categorized as mild, moderate, or severe. Among the 120 participants, 20 (16.7%) reported mild symptoms, 52 (43.3%) reported moderate symptoms, and 48 (40.0%) reported severe symptoms. In Japan, pain severity was categorized into pain-free status, mild, moderate, and severe pain, with no significant differences observed among the study arms [14]. The difference in pain assessment methods and levels of awareness in Japan might explain the divergent results observed between our study and theirs.

In our clinical study, we evaluated PA, OPG, occlusal view, and CBCT images. However, we predominantly used PA view and OPG due to their affordability and diagnostic capabilities, while CBCT was used sparingly due to its higher cost and limited ability to detect radiographic signs of PARLs [15]. A study conducted in the United States focused on the accuracy of different imaging methods, including CBCT, OPG, and MRI, in detecting endodontically treated and untreated teeth. Their findings differ from ours, as our study primarily utilized PA radiographs, while the technologically advanced countries in the American study have better access to advanced imaging modalities [16].

The types of radiographic lesions associated with pulpitis were also examined in our study. The highest incidence in our population was observed in cases of widened PDL space and PA granulomas without root resorption, while the incidence of PA cysts and root resorption was lower. The Belarusian and Croatian populations exhibited similar prevalence rates as ours, with widened PDL and PA granulomas. Conversely, the Russian and Portuguese populations had the lowest prevalence rates, with a higher incidence of cysts and root resorption. These differences may be attributed to various factors specific to each population [17].

Finally, tooth wear was assessed among the study participants, with 21 (17.5%) reporting its presence and the majority (99, 82.5%) not reporting any tooth wear. Statistical analysis revealed a significant association between the presence of tooth wear and the variables of interest. In a study conducted in Japan with 244 participants, 130 (88.7%) reported no attrition, while 14 (10.1%) experienced attrition. Similar to our findings, Japan demonstrated a lower incidence of attrition associated with PARLs [18].

## Conclusions

In this cross-sectional study, PA views were the most frequently performed radiographic view, followed by occlusal views. The most prevalent radiographic lesion was the widening of the PDL space, followed by *PA granuloma* and *PA cyst*. The most common diagnosis was *pulpitis*, followed by *irreversible pulpitis with apical periodontitis*. Factors such as education level, swelling, pus discharge, medication history, and tooth wear were significantly associated with the final diagnoses.

These findings provide valuable insights into the frequency and types of PARLs associated with pulpitis and emphasize the importance of considering various clinical factors in the diagnosis and management of these conditions. Further research and larger studies are warranted to validate and expand upon these findings.
